# Thermospheric parameters contribution to the formation of Yakutsk F_2_-layer diurnal summer time anomaly

**DOI:** 10.1038/s41598-022-17691-1

**Published:** 2022-08-05

**Authors:** Andrey V. Mikhailov, Loredana Perrone

**Affiliations:** 1 Pushkov Institute of Terrestrial Magnetism, Ionosphere and Radio Wave Propagation (IZMIRAN), Troitsk, Moscow, 108840 Russia; 2 Istituto Nazionale di Geofisica e Vulcanologia (INGV), 00143 Rome, Italy

**Keywords:** Space physics, Space physics

## Abstract

The role of thermospheric neutral composition in the formation of the Yakutsk diurnal summer time foF_2_ anomaly is analyzed. Ionospheric stations inside and outside the anomaly area are considered. The effect of neutral composition in foF_2_ is the most noticeable around noontime hours. The difference between observed noontime foF_2_ in two areas is significant at the 99.9% confidence level both for monthly median and individual days. The inferred from ionosonde observations and Swarm neutral gas density thermospheric parameters indicate a significant difference between two areas. The inferred exospheric temperature, Tex at Magadan (inside the anomaly area) is significantly larger than Tex at Tunguska (outside the anomaly area). On the contrary, the inferred atomic oxygen [O] at Tunguska is significantly larger than at Magadan. Different [O] abundance in the two areas is the main reason of the observed difference in noontime foF_2_ values. Vertical plasma drift depending on magnetic declination, D is the only process responsible for the difference between nighttime foF_2_ at Tunguska and Magadan. A possible mechanism of the revealed difference in thermospheric parameters inside and outside the anomaly area is discussed.

## Introduction

Historically F_2_-layer diurnal summer time anomaly is defined an excess of midnight foF_2_ over noontime values. As far as this is known the effect was firstly observed by Bellchambers and Piggott^1^ in 1958 at Halley Bay (76°S, 26°W, dip 64.6°). Similar results were confirmed using observations at Port Lockroy^2^. Later analyzing Antarctic ground-based ionosonde observations it was found^3^ that this effect took place in the area of Weddell Sea and since then it is called Weddell Sea Anomaly. However using TOPEX TEC observations it was shown^4^ how large the anomaly area was in reality situated west of the Faraday ionosonde station over the Bellinghausen Sea, so the correct name should be Bellinghausen Sea Anomaly. Similar area with abnormal foF_2_ diurnal variations is located in the Northern Hemisphere around Yakutsk (62.0°N, 129.6°E, dip = 75.4°). Sato^5^ perhaps was one of the first who mentioned this fact. Later a detail morphological analysis of Yakutsk foF_2_ abnormal variations was done by Mamrukov^6^.

A mechanism for such foF_2_ diurnal variations has been proposed immediately^7–9^. In summer at middle and higher latitudes F_2_-region is sunlit practically 24 h and fresh plasma is produced even during nighttime. Upward plasma drift generated by equatorward thermospheric wind during nighttime hours uplifts F_2_-layer from the area of strong recombination. This results in accumulation of plasma at F_2_-region heights increasing foF_2_. The authors stressed: “The ‘evening enhancements’ and ‘midnight maxima’ of foF_2_ which occur over *certain* regions of the Earth in summer are shown to be caused almost entirely by neutral-air winds” and also: “There seems little doubt that the diurnal variation at Port Lockroy is produced, as Kohl & King^7^ suggested, by vertical drifts of ionization.” Of course, the larger station latitude the later is sunset in summer and the later nighttime foF_2_ maximum occurs bearing in mind that equatorward wind maximizes around midnight. Along with this the authors correctly and carefully stressed “over *certain* regions of the Earth”. This is due to the fact that not all stations located at the same latitudes (i.e. subjected to the same solar ionization) manifest the nighttime foF_2_ maximum. The authors did not take into account that vertical plasma drift W depends both on meridional Vnx and zonal Vny components of thermospheric wind W = (Vnx cosD − Vny sinD) sinI cosI, where I and D—inclination and declination of the Earth’s magnetic field. It should be stressed that the effect of zonal wind (via magnetic declination D) on the F_2_-layer had been already discussed that time^10^.

Since then many mechanisms (some of then are purely speculative) have been suggested to explain the diurnal foF_2_ anomaly but the initial idea that evening foF_2_ enhancement and midnight maximum are due to the upward plasma drift under direct solar ionization may be considered as commonly accepted^4,11–14^. Usually the magnitude of diurnal anomaly is estimates by the ratio r = (foF_2_)_00LT_/(foF_2_)_12LT_^6,12,15^. It means that r depends not only on the midnight foF_2_ enhancement but also on the noontime depression and the involved processes may be different due to the difference formation mechanisms of daytime and nighttime F_2_-layer. This is quite different level of analysis—not a morphological but a physical one. The majority of analyses devoted to the foF_2_ diurnal anomaly are done at the morphological level. The physical level needs knowledge of aeronomic parameters responsible for the F_2_-layer formation—first of all thermospheric parameters, solar EUV ionizing radiation and vertical plasma drifts related to thermospheric winds. Attempts to use blindly global empirical models like MSIS and HWM93 without any external control have been undertaken^13,16^. The aeronomic parameters should be consistently related but this consistency is questionable keeping in mind how these empirical models were derived. An attempt to use a first-principle (physical) GSM TIP model in a comparison with top-sounder IK-19 observations gave unsatisfactory results^15^. Unlike the observed with IK-19 position of anomaly centered to ~ 150°E with r ~ 1.5 the calculated anomaly is centered to ~ 80°–90°E with r ~ 1.2 (their Fig. 6). At 150°E the calculated r ~ 0.7, i.e. less by two times compared to the observed one. Later in our paper it is shown that Tunguska station located at 90.0°E does not manifest any diurnal foF_2_ anomaly. It means that the mechanism of Yakutsk foF_2_ diurnal anomaly should be specified in the part of thermospheric parameters contribution. Such analysis as far as we know has not been undertaken before.

The aims of our paper may be formulated as follows.

1. To consider noontime monthly median foF_2_ for ionosonde stations located inside and outside the Yakutsk magnetic anomaly to check whether they are statistically different.
2. To retrieve from ionosonde noontime observations a consistent set of the main aeronomic parameters responsible for the F_2_-region formation to check whether the thermospheric parameters are different for the stations inside and outside the anomaly area using for this comparison Swarm neutral gas density observations.
3. To show the controlling role of thermospheric neutral composition in the observed difference of noontime foF_2_ inside and outside the anomaly area.
4. To check whether nighttime foF_2_ maximum inside the anomaly area and the absence of such maximum outside the anomaly area are totally due to different vertical plasma drifts in the two regions.

### Observations

**Figure 1:**
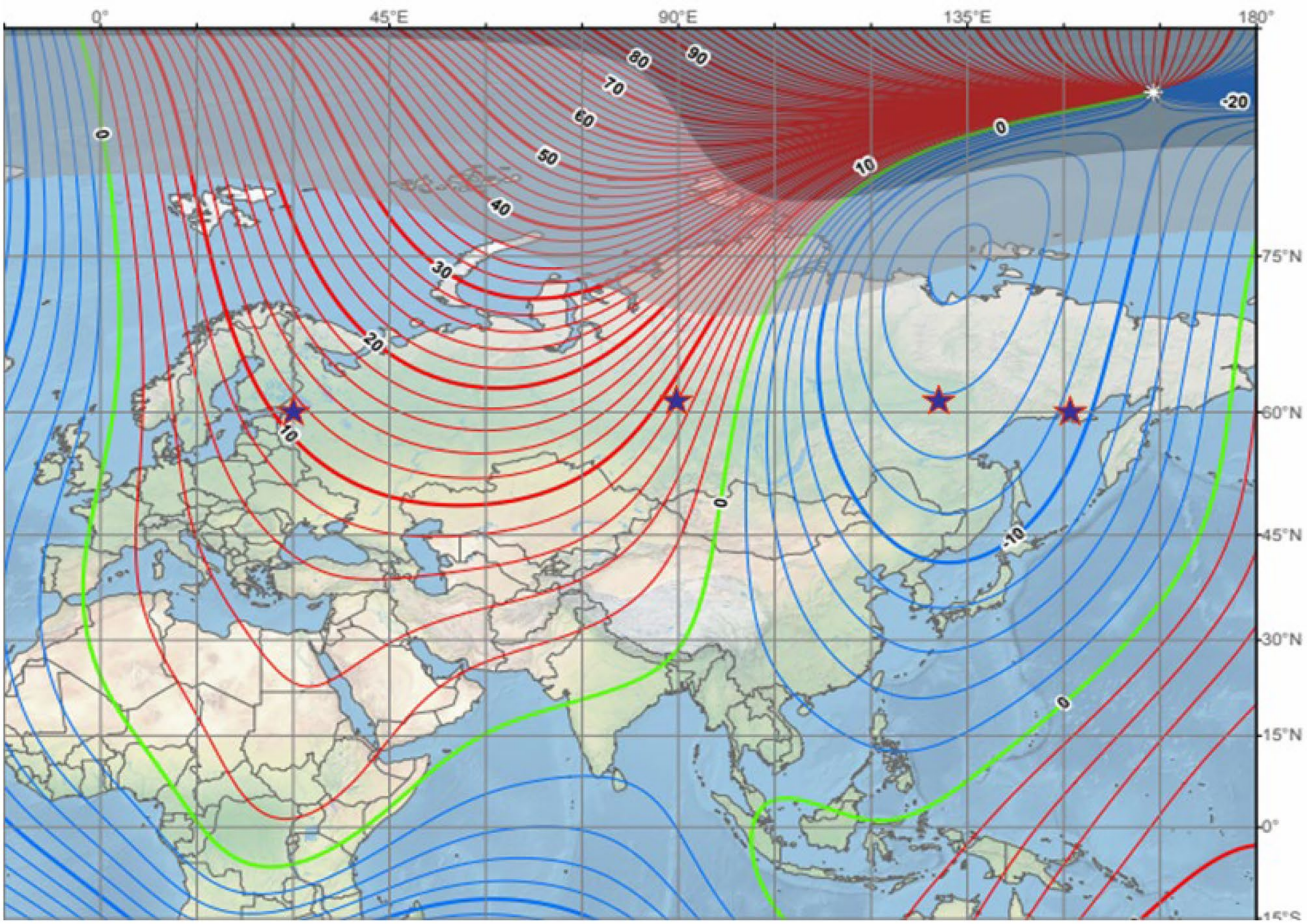
Magnetic field declination, D and analyzed ionosonde stations (asterisks).

The Yakutsk ionospheric anomaly is undoubtedly related to the geomagnetic anomaly located in this area. Fig. 1 exhibits a map of the Earth’s magnetic field declination, D (https://www.ngdc.noaa.gov/geomag/WMM/image.shtml) along with ionospheric stations selected for our analysis: Magadan (60.1°N, 151.0°E, Φ = 50.7°, I = 71.0°, D = −8.3°), Yakutsk (62.0°N, 129.5°E, Φ = 51.0°, I = 75.4°, D = −11.9°), Tunguska (61.6°N, 90.0°E, Φ = 50.7°, I = 77.5°, D = 7.5°), and St. Petersburg (60.0°N, 30.7°E, Φ = 56.2°, I = 72.6°, D = 5.1°), where Φ—magnetic latitude, I—magnetic inclination, and D—magnetic declination. The ionospheric stations have similar geodetic latitudes ~ 61°N therefore they are subjected to same solar illumination and three of them (Magadan, Yakutsk, and Tunguska) have close magnetic latitudes Φ ~ 51°, while they have different magnetic declination, D—negative (westward) at Magadan and Yakutsk and positive (eastward) at Tunguska and St. Petersburg.

**Figure 2:**
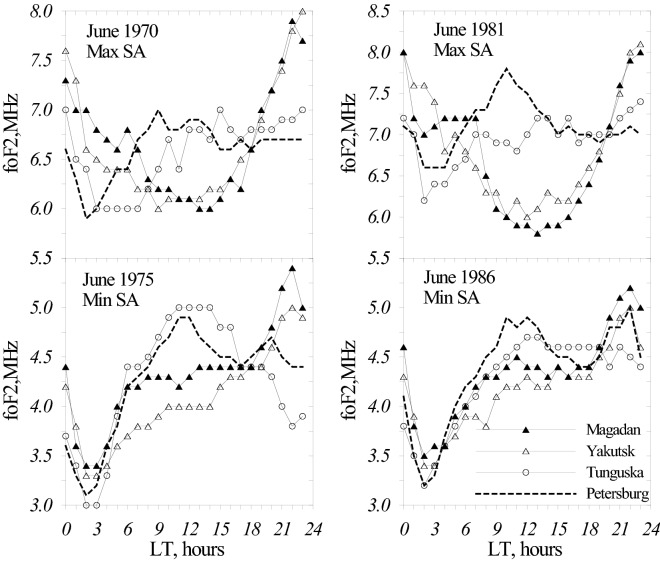
June monthly median foF_2_ diurnal variations at ionospheric stations located in the anomaly area (Yakutsk, Magadan) and outside this area (Tunguska, St. Petersburg) under solar maximum (1970, 1981) and solar minimum (1975,1986) conditions.

Figure 2 gives June monthly median foF_2_ diurnal variations at ionospheric stations located in the anomaly area (Yakutsk, Magadan) and outside this area (Tunguska, St. Petersburg) under solar maximum (1970, 1981) and solar minimum (1975,1986) conditions. Historical foF_2_ observations used in our paper were mainly taken from SPIDR while recent observations—directly from the ionospheric stations. A well-pronounced difference (also mentioned in earlier publications) in foF_2_ diurnal variations is seen in June for the two groups of stations both under solar maximum (1970 monthly F_10.7_ = 154.9, and 1981, F_10.7_ = 156.9) and solar minimum (1975, F_10.7_ = 69.7; 1986, F_10.7_ = 67.6). Inside the anomaly area (Yakutsk, Magadan) maximum in foF_2_ diurnal variations occurs in the vicinity of midnight while outside this area it takes place around noontime.

Figure 2 manifests that stations inside the anomaly area are distinguished not only by larger nighttime foF_2_ but also by lower foF_2_ daytime values. The latter feature was only mentioned in some publications^12^ without any its detail analysis. However this difference may have a fundamental meaning as daytime mid-latitude foF_2_ directly reflects the state of the surrounding thermosphere and the observed difference in foF_2_ may indicate the peculiarities in thermospheric parameters inside the anomaly area.

Let us check if low foF_2_ inside the Yakutsk anomaly is an inalienable feature of this area.

**Figure 3:**
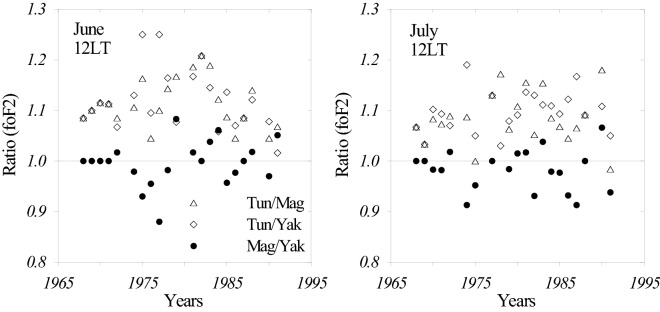
Noontime June and July monthly median foF_2_ ratios for Tunguska/Magadan (triangles), Tunguska/Yakutsk (diamonds), and Magadan/Yakutsk (circles) calculated over the (1968–1991) period.

Figure 3 gives foF_2_ ratios for Tunguska (outside the anomaly area) to Magadan and Yakutsk located inside the anomaly area. The Magadan to Yakutsk ratio is given for a comparison.

We give ratios rather than observed foF_2_ to remove by this way solar cycle variations and to make the plot more visual. Figure 3 shows that Tunguska manifests larger noontime foF_2_ compared to Magadan and Yakutsk while the Magadan/Yakutsk ratio is centered around unity. Therefore one may expect different thermospheric parameters inside and outside the anomaly area.

## Method

Our method^17^ to retrieve thermospheric parameters from ionospheric observations was applied to June-July monthly median foF_2_ and foF_1_ simultaneously observed at Tunguska/Magadan and Tunguska/Yakutsk stations. The number of coinciding years varied from 15 to 18 during the (1968–1991) period with available observations. The method has some versions depending on available observations. The basic version uses observed noontime f_o_F_2_ and plasma frequencies at 180 km height, f_180_ for (10, 11, 12, 13, 14) LT, both observations may be taken from SAO files^18^ if a DPS-4 digisonde is installed at the station in question. If we deal with summer monthly median conditions (like in the current paper) then instead of f_180_ five median foF_1_ values are used. An advanced version of our method additionally uses observed neutral gas density as a fitted parameter. Neutral gas density observations with CHAMP, GOCE, Swarm satellites exist for some years and may be used for the analysis. The inclusion of neutral gas density into the retrieval process increases the reliability of the obtained results. In this case the inferred neutral composition ([O], [N_2_], [O_2_] concentrations), temperature T_ex_ along with vertical plasma drift W and total solar EUV ionizing flux are found consistently with the observed neutral gas density. Namely this version of the method using Swarm (https://earth.esa.int/web/guest/swarm/data-access) neutral density observations was used to confirm our conclusions on thermospheric parameter peculiarities in the anomaly area. Daytime neutral density observed in the vicinity of ionosonde station was reduced to 12 LT, 450 km height and the location of ionosonde using the MSISE00 thermospheric model^19^ and the following expression:

$$ \rho_{station} = \rho_{satellite} \frac{{MSISE00_{station} }}{{MSISE00_{satellite} }}. $$

## Results

**Figure 4:**
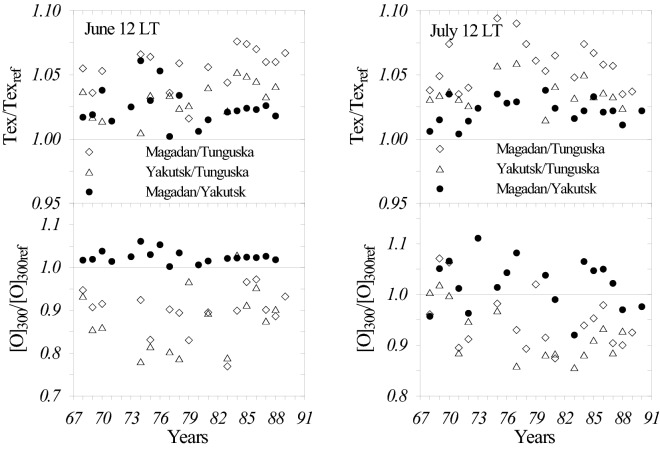
Ratios for exospheric temperature Tex and atomic oxygen [O] at 300 km retrieved at Tunguska, Yakutsk, and Magadan stations for June and July at 12 LT. The subscript ‘ref’ is referred to a station standing in the denominator.

Retrieved thermospheric parameters at Tunguska, Yakutsk, and Magadan stations for June and July 12 LT using coinciding years with available foF_2_ and foF_1_ observations were used to find Tex/Tex_ref_ for exospheric temperature and [O]/[O]_ref_ for atomic oxygen at 300 km ratios given in Fig. 4. The subscript ‘ref’ is referred to a station standing in the denominator.

Figure 4 indicates a tendency for exospheric temperature Tex to be larger at Magadan and Yakutsk located inside the anomaly area compared to Tunguska located outside this area. The inverse situation takes place for atomic oxygen—its concentration is smaller in the anomaly area. The Magadan/Yakutsk ratio is close to unity. This is an interesting result that has not been earlier mentioned in publications devoted to the Yakutsk foF_2_ diurnal anomaly. The difference in atomic oxygen abundance in the two areas explains the observed difference in foF_2_ (Fig. 3) as^20^ NmF_2_ = 1.24 × 10^4^(foF_2_)^2^ ~ [O]^4/3^.

To check and confirm this result available Swarm satellite neutral gas density observations (https://earth.esa.int/web/guest/swarm/data-access) for summer months were analyzed to find coinciding dates with available Magadan and Tunguska foF_2_ and foF_1_ observations. Overall 39 coinciding dates in June–July of 2015–2016 have been found. They were developed with our method^17^ and an example of obtained results is given in Table 1 for Magadan and Tunguska in June 2016.

**Table 1:** Observed noontime NmF_2_ (in 10^5^ cm^−3^), swarm neutral gas density ρ (in 10^–16^ g cm^−3^) reduced to the location of ionosonde, 450 km height, and 12 LT, inferred hmF_2_ (in km), exospheric temperature Tex (in K), atomic oxygen [O] (in 10^8^ cm^−3^) at 300 km, vertical plasma drift W (in m s^−1^), and daily Ap index (in nT).

Magadan, June 2016							Tunguska, June 2016						
Date	NmF_2_	hmF_2_	ρ_450_	Tex	[O]_300_	W	NmF_2_	hmF_2_	ρ_450_	Tex	[O]_300_	W	Ap
01	2.36	242	7.09	1061	2.40	−9.1	3.22	244	6.38	976	2.94	−9.8	5
02	2.40	219	4.13	949	1.99	−12.0	3.89	237	3.82	884	2.45	−8.8	3
03	2.47	231	4.18	966	1.94	−9.1	4.17	239	4.12	880	2.69	−8.8	2
06	2.19	282	6.36	1039	2.31	10.5	2.74	298	5.85	1030	2.11	19.8	23
07	2.30	244	6.98	1050	2.46	−9.2	3.40	245	6.27	958	3.16	−9.9	8
10	2.44	235	4.20	965	1.99	−9.4	3.66	249	4.10	892	2.54	−9.0	6
11	2.29	233	5.64	1027	2.15	−9.5	2.78	241	5.10	961	2.41	−9.3	9
12	2.29	234	4.74	998	1.95	−8.5	2.66	238	4.43	948	2.15	−8.6	10
13	2.27	248	7.91	1077	2.62	−9.2	3.14	248	7.19	1007	3.03	−9.5	8
14	2.47	300	8.17	1109	2.32	18.3	3.14	302	7.84	1067	2.52	20.9	21
15	2.26	239	5.53	1019	2.32	−9.9	2.86	239	4.25	920	2.31	−8.6	11
17	2.33	242	6.66	1042	2.45	−9.2	3.14	246	6.58	979	2.97	−9.6	7
18	2.26	234	5.83	1026	2.20	−10.8	2.98	242	5.91	974	2.69	−9.6	7
19	2.40	238	5.72	1020	2.20	−8.7	3.06	242	5.73	972	2.69	−9.0	4
22	2.58	239	5.75	996	2.42	−9.9	3.57	248	5.88	932	3.13	−8.9	12
23	2.33	242	6.36	1026	2.42	−8.5	3.48	242	5.45	928	2.95	−9.5	10
25	2.26	229	3.50	933	1.78	−7.0	2.78	232	2.94	864	1.96	−9.0	7
26	2.26	235	5.63	1008	2.20	−9.8	2.94	241	5.23	943	2.64	−9.8	10
27	2.33	249	6.63	1030	2.51	−7.6	2.58	246	5.89	983	2.59	−8.3	8
28	2.22	225	3.58	945	1.72	−9.0	2.82	232	3.15	875	2.02	−9.3	5
29	2.26	239	4.76	983	2.03	−8.5	2.86	239	4.72	936	2.45	−8.5	4

The analyzed June 2016 period basically was magnetically quiet with two disturbed days on June 06 and June 14 clearly distinguished by inversed (equatorward) thermospheric wind corresponding to positive vertical plasma drift, W resulted in large hmF_2_. All other days manifest a moderate downward plasma drift ~ −9 m/s corresponding to normal poleward daytime thermospheric wind. Observed NmF_2_ at Tunguska are systematically larger than at Magadan similar to earlier given results in Fig. 3. The difference is significant at a confidence level > 99.9% while the difference between inferred hmF_2_ is insignificant at the two stations. In accordance with results in Fig. 4 the inferred Tex at Magadan (average Tex = 1012 K) is significantly (the confidence level > 99.9%) larger than Tex at Tunguska (average Tex = 948 K). On the contrary, the inferred [O] at Tunguska is significantly (the confidence level > 99.9%) larger than at Magadan. These anti-phase Tex and [O] variations result in insignificant difference in the neutral gas density at 450 km observed at the two stations (Table 1). Other analyzed June–July periods demonstrate similar results but they are not given not to overload the paper.

## Discussion

It is well-known that foF_2_ diurnal anomaly is only observed at some stations and it may be absent at other stations located at same latitudes i.e. subjected to same solar illumination (Fig. 2). The formation mechanism of mid-latitude F_2_-layer includes photo-ionization of neutral species (O, O_2_, N_2_), plasma transfer by diffusion and thermospheric winds and its recombination via the chain of ion-molecular reactions. It should be stressed that in summer (June-July) under magnetically quiet conditions (see Table 1) Tunguska, Yakutsk, Magadan with Φ ~ 51.0° are classic mid-latitude stations not subjected to any auroral effects mentioned by the authors^15,21^.

**Figure 5:**
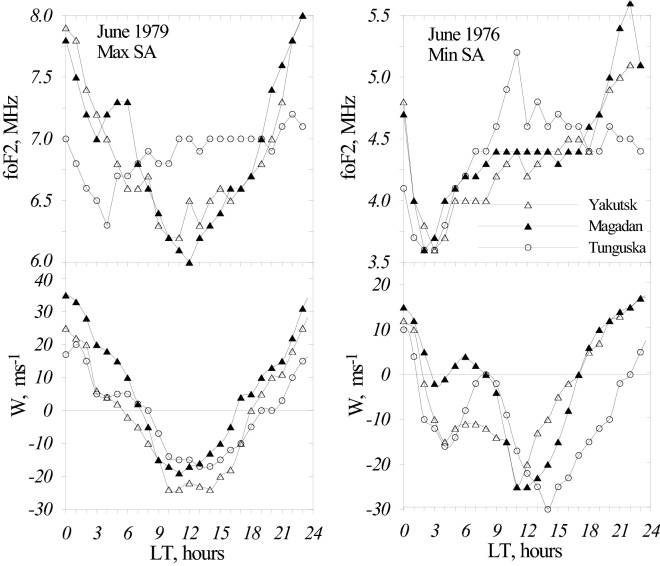
Observed monthly median foF_2_ diurnal variations at Yakutsk and Magadan (stations inside the anomaly area) and Tunguska (a station outside the anomaly area) under solar maximum (1979) and solar minimum (1976) conditions (top panel). Bottom panel—inferred diurnal variations of vertical plasma drift.

Let us check if Tunguska located outside the anomaly area and stations inside the area do manifest different diurnal variations of vertical plasma drift W. This may be done by fitting with W observed diurnal foF_2_ variations. Neutral composition and temperature found for noontime (as this was explained earlier) are used to normalize MSIS-86 model values for all 24 h used in the fitting procedure. Such step is justified for quiet time and monthly median conditions. By solving the continuity equation for electron concentration in the F_2_-region as this was described^22^ it is possible to find diurnal variations of vertical plasma drift at F_2_-layer heights. Fitting observed foF_2_ diurnal variations with W under non-stationary conditions requires taking into account the pre-history of W variations and special methods are needed to specify W values for previous 5–7 h which contribute to the current foF_2_ value. The results are given in Fig. 5 for monthly median conditions under solar maximum (June 1979) and solar minimum (June 1976).

Figure 5 shows that after noontime W at Tunguska is systematically less (more negative) than at Magadan and this results in lower evening-nighttime foF_2_ as F_2_-layer remains at lower heights with stronger recombination rate. Basically Southward Vnx increases from daytime to midnight hours at both stations increasing upward W towards midnight hours. But in accordance with the expression W = (VnxcosD − VnysinD)sin IcosI (where Vnx − meridional component of thermospheric wind positive to the South, Vny—zonal component of thermospheric wind positive to the East, D—positive to the East, I—positive in the Northern Hemisphere and the vector of total magnetic field B is downward) vertical drift related to Vny overlaps on W related to Vnx variations. In the evening Vny is directed to the East^23^, therefore at Magadan and Yakutsk where D < 0 vertical drift related to Vny is positive (upward) increasing the total upward W (Fig. 5). On the contrary at Tunguska where D > 0 vertical drift related to Vny is negative (downward) decreasing the total upward W. Therefore the declination D of the Earth’s magnetic field is a controlling parameter responsible for the formation of foF_2_ diurnal anomaly bearing in mind the excess of nighttime foF_2_ over daytime ones as this was earlier stressed in some publications^4,11,12^. Vertical plasma drift is the only process responsible for the difference between nighttime foF_2_ at Tunguska and Magadan (also Yakutsk, Fig. 2). The difference in neutral composition (Table 1) works in the opposite direction decreasing the photo-ionization rate at Magadan and Yakutsk.

The other question—why noontime foF_2_ are different at the stations located inside and outside the anomaly area? Fig. 3 and Table 1 manifest that foF_2_ inside the area (Magadan, Yakutsk) are significantly less than outside (Tunguska) the anomaly area. Our analysis has shown that the main reason for this difference is different atomic oxygen abundance in the two areas and this has been shown for the first time. Along with this vertical plasma drift, W related to thermospheric winds which theoretically can also affect foF_2_ turned out to be the same in two areas. Table 1 after removing the disturbed dates of June 06, 14 gives an insignificant difference in W according to Student t-criterion with average W = −9.2 m/s at Magadan and −9.1 m/s at Tunguska.

This is a new and interesting result. Low [O] in the anomaly area is accompanied by larger Tex, the difference between two regions being significant at the 99.9% confidence level (Table 1). This means that a decrease in the atomic oxygen abundance in the anomaly area is essential as it is not even compensated by larger Tex. Such variations of thermospheric parameters are typical of magnetic storm conditions when disturbed neutral composition with low O/N_2_ ratio and high Tex is transferred from the auroral zone to middle latitudes. But we deal with magnetically quiet conditions (Table 1) and three analyzed stations have close magnetic latitudes Φ ~ 51°, therefore no auroral effects are expected.

Further analysis of this effect has shown that similar variations of Tex and [O] are to some extent reflected in the empirical MSISE00 model^19^. The model was run for the same dates with Swarm observations in June–July (2015–2016). The results are similar to shown in Table 1. MSISE00 gives Tex which is systematically larger and [O]_300_ which is systematically less at Magadan compared to Tunguska. However on average our inferred [O]_Tun_/[O]_Mag_ ratio is by ~ 7% larger than MSISE00 predicts.

Some comments can be done in relation to the dependence on solar activity. We took June 1970 as a period of solar maximum with 3-month F_10.7_ = 158.4 and June 1975 for solar minimum with 3-month F_10.7_ = 72.3. Observed monthly median foF2 and retrieved thermospheric parameters in a comparison with MSISE00 thermospheric model values are given in Table 2.

**Table 2:** Ionospheric and thermospheric parameters inside (Magadan) and outside (Tunguska) the anomaly area in June under maximum (1970) and minimum (1975) solar activity.

Stations	Periods	foF2, MHz	[O]_300_ × 10^8^ cm^−3^	Tex, K	W, m s^−1^	[O]_300_ × 10^8^ cm^−3^ MSISE00	Tex, K MSISE00
Tunguska	1970	6.8	6.35	1244	−13.6	6.10	1223
	1975	5.0	2.60	867	−12.6	2.51	842
Magadan	1970	6.1	5.82	1310	−13.0	5.48	1259
	1975	4.3	2.16	923	−13.9	2.31	883

MSISE00 model values are given for a comparison.

In accordance with earlier mentioned results Table 2 shows that observed foF_2_ at Tunguska are larger than at Magadan both under solar maximum and minimum but ratio max/min is about the same ~ 1.4 at the two stations. Similar solar activity variations manifest inferred exospheric temperature Tex with max/min ratio ~ 1.42 and this is very close to MSISE00 ratio ~ 1.44. Both retrieved and model [O]_300_ demonstrate similar solar cycle variations with max/min ratio ~ 2.6 and ~ 2.4, correspondingly. This is an interesting result as MSISE00 has nothing common with the retrieval process^17^. In contrast to thermospheric parameters vertical plasma drift W related to neutral winds manifests no solar activity variations being ~ −13 m/s both under solar maximum and minimum. The absence of solar activity variations for thermospheric winds was taken into account in the global empirical model^23^.

The revealed regional difference in thermospheric parameters may be explained in the framework of global longitudinal variations of ionospheric and thermospheric parameters^24^.

Historically the mechanism of longitudinal variations in neutral composition and temperature has been associated with high-latitude heating and displacement between the geomagnetic and geographic poles^25–27^. The near-to-pole American longitudinal sector manifests larger [N_2_] and lower [O] concentrations compared to the European (far-from-pole) sector at the same geographic latitudes. It was suggested that June auroral heating was systematically larger in the American sector due to the larger conductivity in the auroral zone^24^.

## Conclusions

The obtained results may be formulated as follows.

1. June–July noontime foF_2_ is systematically less inside the Yakutsk anomaly area (Magadan, Yakutsk stations) than outside this area (Tunguska station) increasing by this way the magnitude of foF_2_ diurnal anomaly. The difference in foF_2_ between two areas is significant at the 99.9% confidence level both for monthly median and individual days. The observed difference in foF_2_ directly indicates the difference in thermospheric parameters in the two areas.
2. The inferred from ionosonde observations thermospheric parameters indicate a significant difference between two areas. Swarm neutral gas density observations were used in the retrieval process. The inferred Tex at Magadan is significantly (the confidence level > 99.9%) larger than Tex at Tunguska. On the contrary, the inferred [O]_300_ at Tunguska is significantly (the confidence level > 99.9%) larger than at Magadan. This means that a decrease in the atomic oxygen abundance in the anomaly area is essential as it is not compensated by larger Tex. These anti-phase Tex and [O] variations result in insignificant difference in the neutral gas density at 450 km observed in the two areas.
3. Different atomic oxygen abundance in the two areas is the main reason of the observed difference in noontime foF_2_ values. Along with this noontime vertical plasma drift, W related to thermospheric winds, which theoretically can also affect foF_2_, turned out to be the same in two areas.
4. Vertical plasma drift related to thermospheric winds is the only process responsible for the difference between nighttime foF_2_ at Tunguska and Magadan (also Yakutsk). The difference in atomic oxygen and temperature works in the opposite direction decreasing the photo-ionization rate inside the anomaly area. It is confirmed that the declination D of the Earth’s magnetic field is a controlling (via zonal thermospheric wind, Vny) parameter responsible for the formation of foF_2_ diurnal anomaly bearing in mind the excess of nighttime foF_2_ over daytime ones.
5. The revealed difference in thermospheric parameters inside and outside the anomaly area may be considered in the framework of global longitudinal variations in the thermosphere associated with high-latitude heating and displacement between the geomagnetic and geographic poles.

## Data Availability

In this study, we used the following observational data ionosonde data from SPIDR (Space Physics Interactive Data Resource) http://spidr.ionosonde.net/spidr, from Swarm accessible from (https://earth.esa.int/web/guest/swarm/data-access).
